# Age-period-cohort effect on motor neuron disease mortality in the United States, 2001–2020

**DOI:** 10.3389/fneur.2025.1751690

**Published:** 2026-01-21

**Authors:** Jaime Raymond, Theodore Larson, Theresa Nair, John Kaufman, D. Kevin Horton, Marc Weisskopf, Suraya Mohidul, Paul Mehta

**Affiliations:** 1 Office of Innovation and Analytics, Agency for Toxic Substances and Disease Registry (ATSDR)/Centers for Disease Control and Prevention (CDC), Atlanta, GA, United States; 2 Department of Environmental Health, Harvard T.H. Chan School of Public Health, Boston, MA, United States; 3 Hite Consulting Inc., Atlanta, GA, United States

**Keywords:** age-period-cohort, ALS, amyotrophic lateral sclerosis, APC, MND, mortality, motor neuron disease

## Abstract

**Introduction:**

Motor neuron diseases (MND) are progressively fatal diseases causing loss of motor neurons throughout the body. Recent studies have suggested an increase in prevalence and mortality of amyotrophic lateral sclerosis (ALS), the most common adult-onset MND. It is unclear whether the increase is because of earlier diagnosis or potentially new exposures. Age-period-cohort (APC) analysis can help identify contributors to temporal disease trends by differentiating impacts of biological aging, historical time period, and birth cohort. The aim of this study is to evaluate APC effects on MND mortality in the United States from 2001 to 2020.

**Methods:**

We analyzed deaths by MND for the period 2001–2020 in subjects aged 40–84 years. We used APC modeling to compute net drift, local drift, longitudinal age curve, rate ratios (RR), and confidence intervals (CI) for each period and cohort. Analysis used the APC Web Tool provided by the United States’ National Cancer Institute.

**Results:**

Over the 20-year period, there were 119,890 MND deaths. Men consistently had higher mortality compared to women. Analysis yielded noteworthy birth cohort effects for men. For men, the cohort RR decreased from 1919 to 1953 and peaked again between 1959 and 1963. Men born after 1973 had a reduced RR = 0.77 (95% CI = 0.63–0.94). Women born after 1973 had a cohort RR = 1.00 (95% CI = 0.75–1.34).

**Conclusion:**

APC analysis revealed potentially impactful age, period, and cohort effects in U.S. MND mortality between 2001 and 2020, with higher mortality among men and evidence of sex-specific cohort patterns. Cohort effects suggest potential generational differences in risk. Further investigation is needed to disentangle ascertainment effects from true etiologic influences.

## Introduction

Motor neuron disease (MND) is the term for rapidly progressive neurodegenerative disorders that affect upper and lower motor neurons in the body. Amyotrophic lateral sclerosis (ALS) is the most common form of adult-onset MND, comprising approximately 79% of MND deaths (1). While considered rare, the incidence rates of MND range from 0.4 to 5.6 per 100,000 person-years worldwide per year (2–5). The incidence of MND increases with age. It peaks between 65 and 75 years and then decreases (2, 6). Recent studies indicate a notable increase in the incidence and mortality rates for MND and ALS in the United States (7–10). However, it is unclear whether these increases reflect a rise in the cases or are due to improved case ascertainment or awareness by clinicians. The increase in MND mortality rates has been seen globally (11, 12).

Familial ALS, a hereditary form of MND, accounts for 5–10% of cases. The remaining cases have no clearly defined etiology (13). Several studies found MND and ALS to be more common in men (14–16) and those with military service history (17). Motor neuron degeneration in non-hereditary ALS is likely a multifactorial process, consisting of both genetic and environmental factors (18, 19). Research indicates that epigenetics plays a significant role in the development of MND (20–22). Relatedly, the increase in the incidence and mortality of MND could be due to various environmental or occupational risk factors that evolved over time.

Due to the complexity of this disease, the federal Agency for Toxic Substances and Disease Registry (ATSDR), working with the Centers for Disease Control and Prevention (CDC), established the National ALS Registry (Registry). The Registry evaluates the public health burden of ALS in the United States (14, 23). Congress mandated the Registry to determine the epidemiology of ALS (incidence, prevalence, mortality), assess case demographics, and evaluate risk factors and possible etiologies (24).

Age-period-cohort (APC) models are statistical tools used in epidemiology to unravel the effects of age, time period, and birth cohort on health outcomes, particularly mortality rates associated with chronic diseases (25, 26). Age effects are variations in mortality rates that occur due to biological and social processes associated with aging. Period effects are changes in mortality rates that occur due to external factors that affect all age groups at a specific calendar time (e.g., year). Cohort effects arise from the unique experiences or exposures of a specific birth cohort as they progress through life. Understanding how these three factors interact and influence disease incidence and mortality over time can help public health professionals develop targeted public health interventions for different populations. To our knowledge, APC models have not been used to study MND mortality in the United States. This study evaluates APC effects on MND mortality in the United States from 2001 to 2020.

## Methods

### Data source

We used the most recent 20-year period of mortality rates for MND derived from 2001 to 2020 death certificate data from the CDC Wide-Ranging Online Data for Epidemiologic Research (CDC Wonder). MND cases were defined using diagnostic death code G12.2 under the International Classification of Diseases, 10th edition (ICD-10). To be included in the initial listing of MND deaths, G12.2 must have appeared in the underlying or multiple causes of death fields. Although an ICD-10 code exists for ALS (G12.21), it is a clinical code and not used for cause of death. CDC Wonder are only available with a maximum of four characters (e.g., G12.2), so this analysis was limited to MND deaths and not ALS deaths specifically. To determine the ages to be included in the analysis, we referred to published mortality rates of MND/ALS. U.S. ALS mortality rates are extremely low before age 40 (1) and ALS mortality decreases sharply after age 84 (1). In CDC Wonder, there were few submitted deaths below 40 (1.2% of deaths) and above 84 years of age (6.7% of deaths). Combining the current CDC Wonder data with the previously published analysis of ALS mortality rates, we restricted the analyses to 40–84 years. We obtained sex-specific population mortality rate denominators in 5-year increments from the National Center for Health Statistics (27).

### Statistical analysis

To analyze the temporal trends of MND, we used join-point regression models, a robust statistical method employed to identify significant changes in trends over time. Typical regression models do not work for temporal analysis because age, period, and cohort are perfectly correlated (period = age + cohort). This collinearity renders standard regression models statistically invalid for isolating independent age, period, or cohort effects (28). APC analysis is particularly useful for analyzing health data, such as that obtained from CDC Wonder. To apply the APC analysis using a join-point regression model, the dataset was organized into four 5-year periods (2001–2005, 2006–2010, 2011–2015, and 2016–2020) and nine 5-year age groups from 40–44 to 80–84 years old. This resulted in 11 birth cohorts from 1919–1923 to 1969–1973. For each age group and 5-year period, we computed sex- and age-specific death rates. Sex-specific analysis was chosen because MND incidence rates, and therefore mortality rates, differ by sex: males had a slightly higher rate than females. We assessed MND deaths using the free, publicly available APC modeling tool developed by the U.S. National Cancer Institute (29). This tool provides built-in functionality to fit models and conduct hypothesis testing.

To assess trends in MND mortality, we estimated age-specific longitudinal rates, period- and cohort-specific rate ratios (RR), local drifts, and net drift. Local drifts indicate annual percentage changes in MND mortality rate for each age group, essentially how the mortality rate for a particular age group changes each year, after adjusting for period and cohort effects. In contrast, net drifts represent the overall annual percentage change in MND mortality rate across all groups. Age-specific longitudinal rates reflect how MND mortality rates change over time for specific age groups. The period and cohort-specific RRs compare MND mortality rates across different time periods and birth cohorts, providing insights into temporal trends and generational differences. When exact numbers could not be used, we used central age group, calendar year, and birth cohort as reference points within each group. We used Wald Chi-square tests to determine the statistical significance of the change. The threshold for statistical significance was set at *p* < 0.05 for all two-sided tests. We interpret RRs 1.2 to 1.5 as showing a weak association and greater than 1.5 as showing a moderate to strong association.

## Results

**Figure 1:**
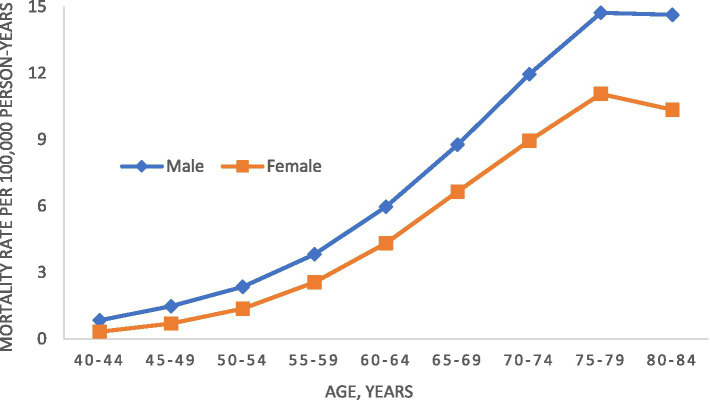
Age-specific motor neuron disease mortality rate in the United States, 2001–2020.

From 2001–2020, there were 119,890 deaths from MND (65,693 men and 54,197 women) among people 40–84 years of age. The overall adjusted mortality rate was 3.8 MND deaths per 100,000 person-years. Age-specific male and female mortality rates for 2001 to 2020 are shown in Figure 1. For both men and women, mortality rates peaked between ages 75 and 79 years. Both observed a decline in rates afterwards, although men to a lesser degree. For all ages, mortality rates were higher in men than women. During the study period overall, men had an age-adjusted mortality rate of 4.2 (95% CI, 4.2–4.3) per 100,000 person-years while women had an age-adjusted mortality rate of 2.9 (95% CI, 2.9–3.0).

### Age by cohort effects

**Figure 2:**
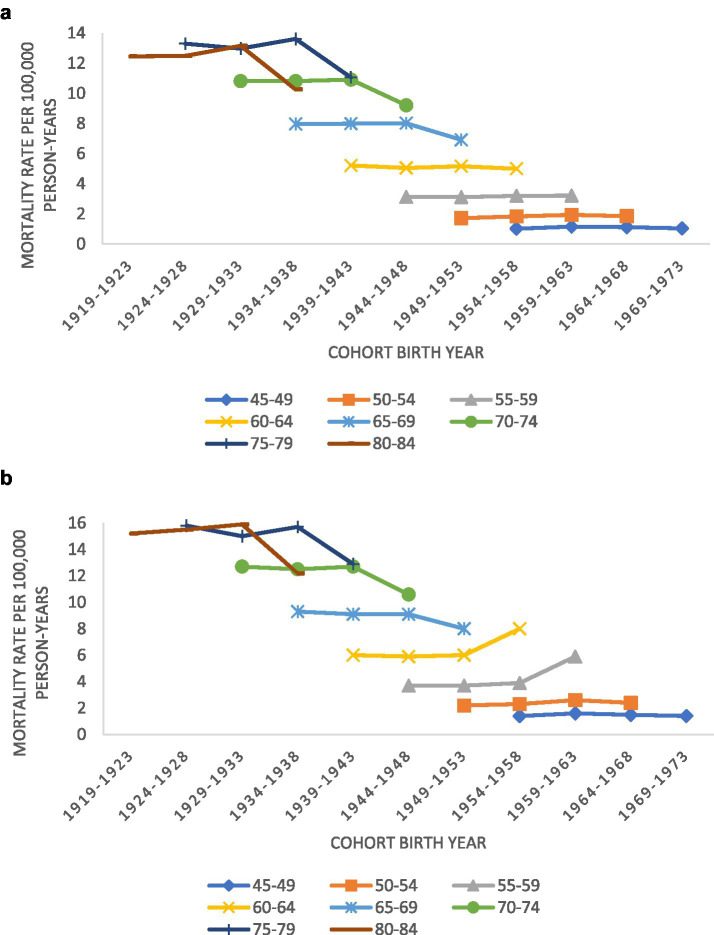
**(A)** Trends in motor neuron disease mortality rates for women by age groups according to year of birth (1919–1973) in the United States; **(B)** trends in motor neuron disease mortality rates for men by age groups according to year of birth (1919–1973) in the United States.

Figures 2A,B display MND mortality rate trends in the United States, for men and women by age group and birth cohort (birth years 1919–1973). Both sexes saw a drop in mortality rates for the oldest birth cohort. However, men born between 1954 and 1963 saw a 33–50% increase in MND mortality. Women in the same birth cohort did not see any increase in MND mortality.

### Age effects

**Figure 3:**
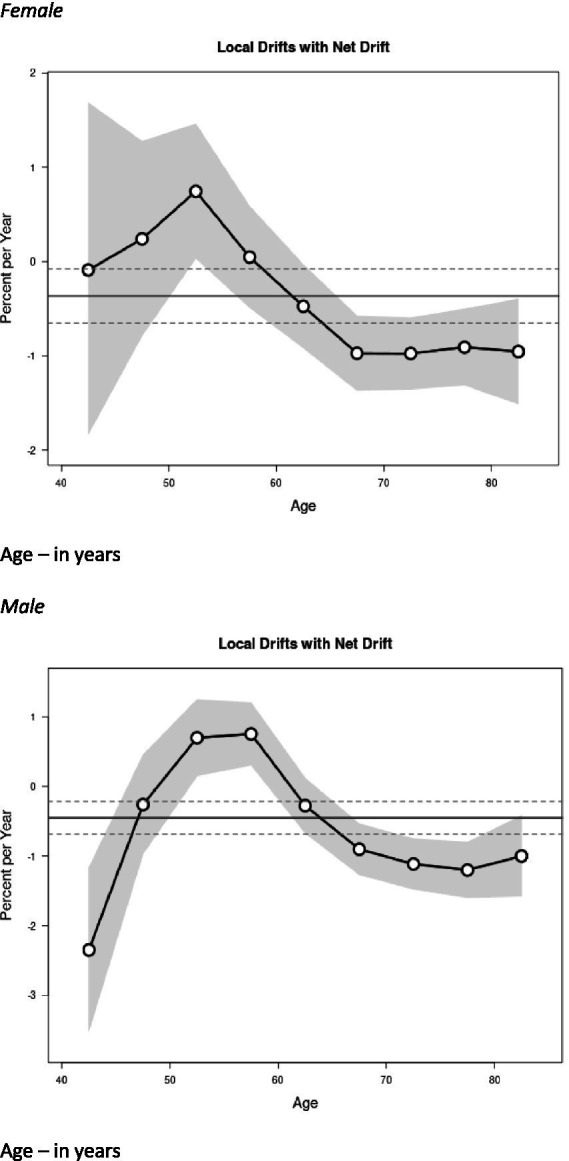
Local drift value of motor neuron disease (MND) mortality rates; age group-specific annual percent change (%) in MND mortality and corresponding 95% confidence intervals (gray area) by sex. Age – in years.

Figure 3 shows the sex-specific net and local drifts. The net drift represents the overall annual percentage change in the age-standardized MND mortality rate across all age groups. The local drifts represent the age-specific annual percentage changes in the MND mortality rates for each individual age group. Local drift values were under 0 in all age groups after age 65 for both sexes. This means that MND mortality rates increased more gradually or decreased compared to ages <65 years. Males had significantly elevated local drift for ages 50–60 years (i.e., experienced the highest percentage increase per year in the MND mortality rate). Women saw elevations slightly earlier, before age 50 years. Local drift values were lowest for men aged 40–44 years.

### Period effects

**Figure 4:**
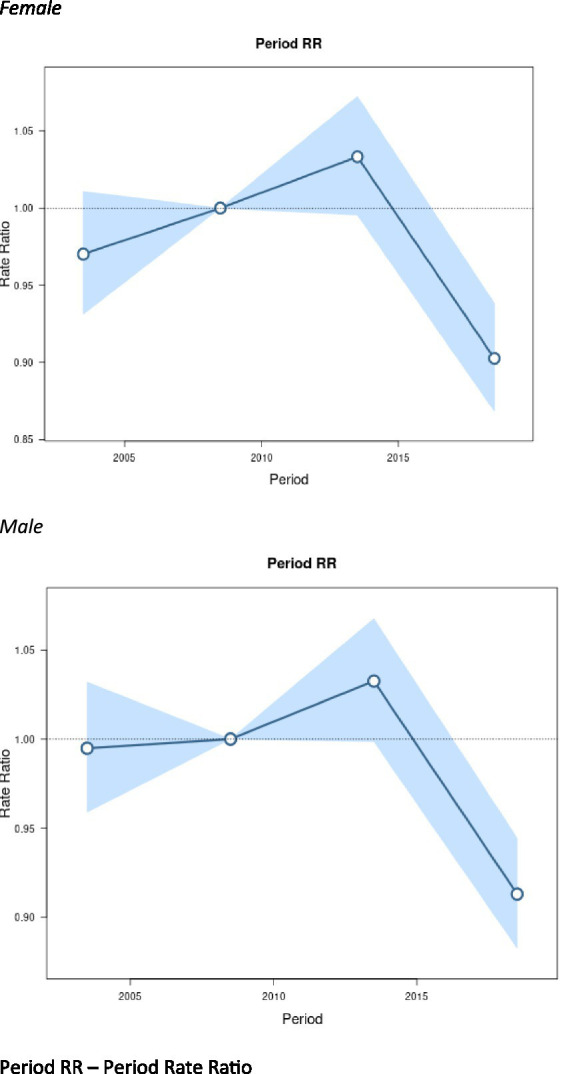
Period effects on motor neuron disease (MND) mortality rates: obtained from age-period-cohort analyses for MND mortality rates and corresponding 95% confidence intervals (blue area) by sex. Period RR – period rate ratio.

Figure 4 displays period effects, which represent the variations in mortality rates over time associated with all age groups. We observed similar period effects by sex. Women had a slightly lower period RR in 2005 compared to men, but the RR was not statistically significant.

### Cohort effects

**Figure 5:**
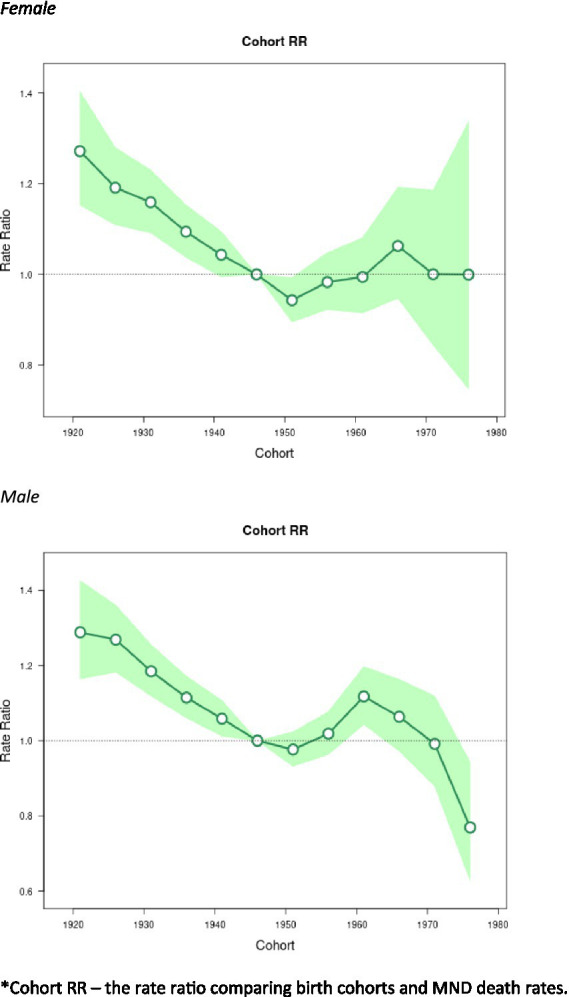
Cohort effects on Motor Neuron Disease (MND) mortality rates: obtained from age-period-cohort analyses for MND mortality rates and corresponding 95% confidence intervals (green area) by sex. *Cohort RR – the rate ratio comparing birth cohorts and MND death rates.

The birth cohort effects associated with changes in mortality rates are represented in Figure 5. The cohort effects showed downward trends from 1920 to 1954 and then flatten out afterwards. From 1955 to 1965, the cohort effects show a slight increase, although the values were not significant. The only statistically significant exception for the cohort effects were males around 1960 (RR = 1.12, 95% CI: 1.04–1.20), after which, there was a general downward trend.

Table 1 summarizes the effects of age, period, and cohort of MND mortality between 2001 and 2020 in the United States. The table highlights differences between men and women across various age groups and time periods. Table 2 displays the Wald Chi Square Tests, local drifts, and net drifts. The data indicate statistically significant cohort and period effects for males. We saw similar results for MND mortality for women regarding cohort, period, and drift.

**Table 1:** Age, period, and cohort effects on motor neuron disease (MND) mortality in the United States, 2001–2020, by sex.

Group	Men		Women	
	Effect	95% CI*	Effect	95% CI
Mortality rate per 100 k p-y^ by age				
40–44	0.85	0.76–0.94	0.32	0.27–0.37
45–49	1.41	1.30–1.54	0.68	0.61–0.76
50–54	2.24	2.10–2.40	1.34	1.24–1.46
55–59	3.70	3.52–3.90	2.60	2.45–2.76
60–64	5.91	5.66–6.17	4.38	4.18–4.60
65–69	8.55	8.22–8.89	6.59	6.32–6.87
70–74	11.10	10.65–11.56	8.41	8.05–8.79
75–79	12.83	12.16–13.54	9.90	9.37–10.48
80–84	12.11	11.35–12.92	8.78	8.21–9.38
Period rate ratio				
2001–2005	0.99	0.96–1.03	0.97	0.93–1.01
2006–2010	1.00		1.00	
2011–2015	1.03	1.00–1.07	1.03	1.00–1.07
2016–2020	0.91	0.88–0.94	0.90	0.87–0.94
Cohort rate ratio				
1919–1923	1.29	1.16–1.43	1.27	1.15–1.40
1924–1928	1.27	1.18–1.36	1.19	1.11–1.28
1929–1933	1.19	1.12–1.26	1.16	1.09–1.23
1934–1938	1.12	1.06–1.17	1.10	1.04–1.15
1939–1943	1.06	1.01–1.11	1.04	0.99–1.10
1944–1948	1.00		1.00	
1949–1953	0.98	0.93–1.02	0.94	0.90–0.99
1954–1958	1.02	0.96–1.08	0.98	0.92–1.05
1959–1963	1.12	1.04–1.20	0.99	0.91–1.08
1964–1968	1.06	0.97–1.16	1.06	0.95–1.19
1969–1973	0.99	0.88–1.12	1.00	0.84–1.19
1974–1978	0.77	0.63–0.94	1.00	0.75–1.34

*CI – Confidence interval.

^100 k p-y – 100,000 person years.

**Table 2:** Age, period, and cohort effects on motor neuron disease (MND) mortality in the United States, 2001–2020, by sex.

Group	Men (Wald Chi square test for estimable functions)	Test statistic *p*-value	Women (Wald Chi square test for estimable functions)	Test statistic *p*-value
Net drift	14.28	0.0002	6.23	0.0126
All period rate ratio	41.28	<0.0001	59.34	<0.0001
All cohort rate ratio	61.06	<0.0001	53.00	<0.0001
All local drifts	61.05	<0.0001	21.98	0.009

## Discussion

Analyzing temporal trends in MND mortality in the United States revealed statistically significant age, period, and cohort effects, some of which differed by sex. This study, covering data from 2001 to 2020, highlights key findings related to MND mortality rates by age and birth cohort. The observed increase in MND death rates, particularly among men born between 1954 and 1963, suggests that specific birth cohorts may be experiencing unique risk factors for MND. The risk factors could be a combination of genetic, environmental, or lifestyle influences that are prevalent in this cohort but not in others. This analysis of MND mortality rates indicates that period effects—which refer to factors that influence the entire population during a specific time frame—may be less important in explaining the temporal trends observed in MND mortality. We are unaware of any other studies of MND that have used APC modeling to understand changes in mortality rates in the United States. However, the approach has been used to examine MND mortality and incidence in other countries (30–32).

Our findings align with other reports on MND mortality trends by sex and age. Men consistently had higher MND mortality rates across all age groups compared to women (1, 6, 15, 33). The age-adjusted mortality rate for men was 3.9 per 100,000 person years compared to 2.7 per 100,000 person years for the study period for persons 40–84 years of age. When examining MND mortality rates by age, for both sexes rates peaked between 75 and 79 years then declined slightly (34, 35).

Over the study period (2001–2020), age-adjusted MND mortality rates remained relatively stable in the United States. However, we found statistically significant differences between birth cohorts independent of their age at death. Birth cohorts include all individuals born at the same time. Several studies have shown differences between men and women regarding MND mortality (1, 4, 8, 11, 35, 36). However, to our knowledge, no study has investigated birth cohorts stratified by sex. Similarly, MND mortality studies that used APC outside the United States did not stratify sex in the birth cohort analysis (4, 9, 36). In our study, there was a slight but statistically significant increase in mortality for the male cohort born between 1954 and 1963. This cohort effect was observed graphically and confirmed statistically through APC analyses (*p* < 0.05). There was no increase for women in the same birth cohort. This male-specific cohort effect suggests that specific generations may have been exposed to environmental and behavioral factors or experiences that influenced their MND mortality risk throughout their lives. Changes in environmental exposure could explain cohort effects for MND mortality. Several studies have shown associations between MND mortality and environmental exposures like well water, heavy metals, and cycad ingestion (34, 37, 38). Behavioral risk factors could also play a role in MND mortality by birth cohort. For example, studies found people who participate in vigorous physical activity or who have a military history have an increased risk of ALS (39–42).

While cohort effects are tied to specific generational experiences, period effects are explained by factors that impact all individuals during a specific time frame, regardless of their age or birth cohort. Our study found similar period effects by sex, with slightly lower period RR for women around 2005. Both sexes peaked around 2015. This suggests broad societal or environmental factors affecting the entire population at a given time had a relatively uniform impact on MND mortality across different age groups. One hypothesized period effect is possible improved identification of MND between 2005 and 2015, particular among older adults and women, resulting in higher observed MND mortality during this period (43). Advances in diagnostic awareness, expanded access to specialty care, and improvements in cause-of-death reporting likely contributed to more accurate recognition and documentation of MND over time (44). These trends reflect a broader shift in the medical landscape rather than a true increase in underlying disease risk. The 2014 ALS Ice Bucket Challenge significantly increased ALS and MND awareness (45). In addition, from 2000 to 2020, the number of specialized centers for MND treatment and research increased from 33 to 97 in the United States (46). The increase in MND mortality in women from 2001 to 2015 could also be due to the growing recognition of MND in female populations, which may have been historically underdiagnosed or misdiagnosed. Our study years include the first year of the COVID-19 pandemic. In 2020, we did see a slight uptick in MND mortality. One study found an increase in MND mortality during the first two years of the COVID-19 pandemic. As more data is released, the pandemic could have a period effect for MND mortality (47).

During the earlier years of the study period, the net drift for MND mortality rates among males showed a slight increase with an annual percentage change of approximately 0.3%. This means the overall trend for male MND mortality is rising at a faster rate over time. Notably, the net drift for males experienced a significant decline between 2014 and 2017: there was an annual percentage change of −5.7%. Potential key drivers for this significant decline could be a reduction in misdiagnosis due to El Escorial revisions (48) as well as improved multidisciplinary care extending survival, indirectly lowering mortality rates (49). Following this period, there was stabilization and a minor increase of 0.4% from 2017 to 2020. In contrast, the net drift for females exhibited a more consistent decline over the same timeframe, 2017–2020. The overall annual percentage change was notably lower than that of males, indicating a more favorable trend in MND mortality rates. For females, the declines were more pronounced in the later years, reflecting a decrease in age-adjusted mortality rates from 2014 onwards. The local drift showed negative values for ages over 65 years in both sexes, but we saw elevated values for ages 50–60 in men. Women had a slightly earlier elevation.

This study on MND mortality has several strengths. The large sample size, long study period, and the statistical approach of APC models provide a robust method for examining temporal trends and generational differences. Next, the quality of the data is consistent throughout the years. CDC Wonder data uses standardized definitions, coding schemes, and processing protocols which are derived from regimented, centralized national data systems across the United States. The study also has limitations. While mortality studies offer opportunities to examine diseases over long periods and in large populations, the data may not capture all cases or may include misclassified cases. The accuracy of death certificates in identifying ALS as a cause of death varies across studies and countries. Studies have shown MND mortality data to be consistent in small countries like Italy (50). However, in the United States, the sensitivity of death certificates for ALS was estimated to between 0.85 and 0.87 in 2010. The estimate indicates moderate reliability but leaves room for misclassification or incomplete reporting of contributing causes of death (33, 51). Several studies have highlighted limitations of using death certificate data for ALS research. For instance, while ALS is often listed as the sole cause of death (46% of cases in one study) (51), other immediate causes such as respiratory failure, cardiovascular disease, and pneumonia are frequently underreported. This incomplete reporting hampers efforts to identify preventable causes of death and improve patient care (33, 51).

## Conclusion

The study employed join-point regression and APC models to analyze temporal trends in MND mortality in the United States from 2001 to 2020. This comprehensive analysis provides insights into MND mortality patterns in the United States, across different age groups, time periods, and birth cohorts stratified by sex. The study supports the notion that there may be causes that could have contributed to MND mortality, affecting successive birth cohorts, particularly males born between 1954 and 1963, for whom we observed cohort effects. The findings support the need for further research into disentangling potential interactions on MND and for the National ALS Registry to continue efforts to determine the public health burden of this devastating disease.

## Data Availability

The datasets presented in this article are not readily available because the data that support the findings of this study are not publicly available due to the privacy risks of the subjects and the policies of the data providers (Centers for Medicare & Medicaid Services, Veterans Health Administration, Veterans Benefits Administration).

## References

[1] Larson TC Kaye W Mehta P Horton DK. Amyotrophic lateral sclerosis mortality in the United States, 2011-2014. Neuroepidemiology. (2018) 51:96–103. doi: 10.1159/000488891, 29990963 PMC6159829

[2] Mehta P Raymond J Punjani R Larson T Han M Bove F . Incidence of amyotrophic lateral sclerosis in the United States, 2014-2016. Amyotroph Lateral Scler Frontotemporal Degener. (2022) 23:378–82. doi: 10.1080/21678421.2021.2023190, 35023792

[3] Stevic Z Kostic-Dedic S Peric S Dedic V Basta I Rakocevic-Stojanovic V . Prognostic factors and survival of ALS patients from Belgrade, Serbia. Amyotroph Lateral Scler Frontotemporal Degener. (2016) 17:508–14. doi: 10.1080/21678421.2016.1195410, 27315438

[4] Seals RM Hansen J Gredal O Weisskopf MG. Age-period-cohort analysis of trends in amyotrophic lateral sclerosis in Denmark, 1970-2009. Am J Epidemiol. (2013) 178:1265–71. doi: 10.1093/aje/kwt116, 24064744 PMC3792730

[5] Rose L McKim D Leasa D Nonoyama M Tandon A Bai YQ . Trends in incidence, prevalence, and mortality of neuromuscular disease in Ontario, Canada: a population-based retrospective cohort study (2003-2014). PLoS One. (2019) 14:e0210574. doi: 10.1371/journal.pone.0210574, 30913206 PMC6435115

[6] Engelberg-Cook ESJ Teixeira da Silva Hucke A Vera-Garcia DV Dagher JE Donahue MH Belzil VV . Prognostic factors and epidemiology of amyotrophic lateral sclerosis in southeastern United States. Mayo Clin Proc Innov Qual Outcomes. (2024) 8:482–92. doi: 10.1016/j.mayocpiqo.2024.07.008, 39323877 PMC11422511

[7] Fang F Valdimarsdottir U Bellocco R Ronnevi LO Sparen P Fall K . Amyotrophic lateral sclerosis in Sweden, 1991-2005. Arch Neurol. (2009) 66:515–9. doi: 10.1001/archneurol.2009.13, 19364937

[8] Sejvar JJ Holman RC Bresee JS Kochanek KD Schonberger LB. Amyotrophic lateral sclerosis mortality in the United States, 1979-2001. Neuroepidemiology. (2005) 25:144–52. doi: 10.1159/000086679, 15990445

[9] Ajdacic-Gross V Schmid M Tschopp A Gutzwiller F. Birth cohort effects in neurological diseases: amyotrophic lateral sclerosis, Parkinson's disease and multiple sclerosis. Neuroepidemiology. (2012) 38:56–63. doi: 10.1159/000334632, 22236983

[10] Longinetti E Fang F. Epidemiology of amyotrophic lateral sclerosis: an update of recent literature. Curr Opin Neurol. (2019) 32:771–6. doi: 10.1097/WCO.0000000000000730, 31361627 PMC6735526

[11] Noonan CW White MC Thurman D Wong LY. Temporal and geographic variation in United States motor neuron disease mortality, 1969-1998. Neurology. (2005) 64:1215–21. doi: 10.1212/01.WNL.0000156518.22559.7F, 15824349

[12] Seljeseth YM Vollset SE Tysnes OB. Increasing mortality from amyotrophic lateral sclerosis in Norway? Neurology. (2000) 55:1262–6. doi: 10.1212/WNL.55.9.1262, 11087765

[13] Mitsumoto H Pioro EP. Amyotrophic lateral sclerosis. Philadelphia: Company, FAD. (1998).

[14] Mehta P Raymond J Zhang Y Punjani R Han M Larson T . Prevalence of amyotrophic lateral sclerosis in the United States, 2018. Amyotroph Lateral Scler Frontotemporal Degener. (2023) 24:1–7. doi: 10.1080/21678421.2023.2245858 37602649

[15] Yamakawa M Dwyer S Song X Statland J. Demographics, clinical characteristics, and prognostic factors of amyotrophic lateral sclerosis in Midwest. Muscle Nerve. (2022) 65:217–24. doi: 10.1002/mus.27450, 34708421 PMC8849587

[16] Raymond J Oskarsson B Mehta P Horton K. Clinical characteristics of a large cohort of US participants enrolled in the National Amyotrophic Lateral Sclerosis (ALS) registry, 2010-2015. Amyotroph Lateral Scler Frontotemporal Degener. (2019) 20:413–20. doi: 10.1080/21678421.2019.1612435, 31131638 PMC6946020

[17] Tai H Cui L Shen D Li D Cui B Fang J. Military service and the risk of amyotrophic lateral sclerosis: a meta-analysis. J Clin Neurosci. (2017) 45:337–42. doi: 10.1016/j.jocn.2017.08.035, 28864407

[18] Van Damme P Robberecht W. Recent advances in motor neuron disease. Curr Opin Neurol. (2009) 22:486–92. doi: 10.1097/WCO.0b013e32832ffbe3, 19593125

[19] Eisen A. Amyotrophic lateral sclerosis is a multifactorial disease. Muscle Nerve. (1995) 18:741–52. doi: 10.1002/mus.880180711, 7783764

[20] Saucier D Registe PPW Belanger M O'Connell C. Urbanization, air pollution, and water pollution: identification of potential environmental risk factors associated with amyotrophic lateral sclerosis using systematic reviews. Front Neurol. (2023) 14:1108383. doi: 10.3389/fneur.2023.1108383, 36970522 PMC10030603

[21] Oskarsson B Horton DK Mitsumoto H. Potential environmental factors in amyotrophic lateral sclerosis. Neurol Clin. (2015) 33:877–88. doi: 10.1016/j.ncl.2015.07.009, 26515627 PMC4646848

[22] Goutman SA Boss J Godwin C Mukherjee B Feldman EL Batterman SA. Occupational history associates with ALS survival and onset segment. Amyotroph Lateral Scler Frontotemporal Degener. (2023) 24:219–29. doi: 10.1080/21678421.2022.2127324, 36193557 PMC10067530

[23] Mehta P Raymond J Nair T Han M Punjani R Larson T . Prevalence of ALS in all 50 states in the United States, data from the national ALS registry, 2011-2018. Amyotroph Lateral Scler Frontotemporal Degener. (2024) 25:687–93. doi: 10.1080/21678421.2024.2358786, 38826088

[24] Authority for congressional ALS registry: Centers for Disease Control and Prevention; 2017. Available online at: https://www.cdc.gov/als/ALSRegistryActPublicLaw.html. (Accessed February 1, 2025).

[25] Clayton D Schifflers E. Models for temporal variation in cancer rates. I: age-period and age-cohort models. Stat Med. (1987) 6:449–67. doi: 10.1002/sim.4780060405, 3629047

[26] Clayton D Schifflers E. Models for temporal variation in cancer rates. II: age-period-cohort models. Stat Med. (1987) 6:469–81. doi: 10.1002/sim.4780060406, 3629048

[27] Centers for Disease Control and Prevention NCfHSNVSS, Mortality 1999–2020 on CDC WONDER Online Database. Data are from the Multiple Cause of Death Files, 1999–2020, as compiled from data provided by the 57 vital statistics jurisdictions through the Vital Statistics Cooperative Program 2021. (2020). Available online at: http://wonder.cdc.gov/mcd-icd10.html. (Accessed February 1, 2025).

[28] Bell A. Age period cohort analysis: a review of what we should and shouldn't do. Ann Hum Biol. (2020) 47:208–17. doi: 10.1080/03014460.2019.1707872, 32429768

[29] Rosenberg PS Check DP Anderson WF. A web tool for age-period-cohort analysis of cancer incidence and mortality rates. Cancer Epidemiol Biomarkers Prev. (2014) 23:2296–302. doi: 10.1158/1055-9965.EPI-14-0300, 25146089 PMC4221491

[30] Tobin K Gilthorpe MS Rooney J Heverin M Vajda A Staines A . Age-period-cohort analysis of trends in amyotrophic lateral sclerosis incidence. J Neurol. (2016) 263:1919–26. doi: 10.1007/s00415-016-8215-z, 27372451

[31] McFarlane R Heverin M Walsh C Hardiman O. Irish amyotrophic lateral sclerosis incidence: age, period, and cohort effects using a partial least squares regression model. Neurology. (2024) 102:e209391. doi: 10.1212/WNL.0000000000209391 38728654

[32] Nakken O Lindstrom JC Tysnes OB Holmoy T. Mortality trends of amyotrophic lateral sclerosis in Norway 1951-2014: an age-period-cohort study. J Neurol. (2016) 263:2378–85. doi: 10.1007/s00415-016-8273-2, 27586392

[33] Larson TC Goutman SA Davis B Bove FJ Thakur N Mehta P. Causes of death among United States decedents with ALS: an eye toward delaying mortality. Ann Clin Transl Neurol. (2023) 10:757–64. doi: 10.1002/acn3.51762, 37000988 PMC10187717

[34] Schwartz GG Klug MG. Motor neuron disease mortality rates in U.S. states are associated with well water use. Amyotroph Lateral Scler Frontotemporal Degener. (2016) 17:528–34. doi: 10.1080/21678421.2016.1195409, 27324739 PMC5152538

[35] Mehal JM Holman RC Schonberger LB Sejvar JJ. Amyotrophic lateral sclerosis/motor neuron disease deaths in the United States, 1999-2009. Amyotroph Lateral Scler Frontotemporal Degener. (2013) 14:346–52. doi: 10.3109/21678421.2013.787629, 23621426

[36] Gordon PH Artaud F Aouba A Laurent F Meininger V Elbaz A. Changing mortality for motor neuron disease in France (1968-2007): an age-period-cohort analysis. Eur J Epidemiol. (2011) 26:729–37. doi: 10.1007/s10654-011-9595-0, 21674216

[37] Spencer PS. Hypothesis: etiologic and molecular mechanistic leads for sporadic neurodegenerative diseases based on experience with Western Pacific ALS/PDC. Front Neurol. (2019) 10:754. doi: 10.3389/fneur.2019.00754, 31417480 PMC6685391

[38] Mitsumoto H Garofalo DC Gilmore M Andrews L Santella RM Andrews H . Case-control study in ALS using the national ALS registry: lead and agricultural chemicals are potential risk factors. Amyotroph Lateral Scler Frontotemporal Degener. (2022) 23:190–202. doi: 10.1080/21678421.2021.1936556, 34137650

[39] Chio A Calvo A Dossena M Ghiglione P Mutani R Mora G. ALS in Italian professional soccer players: the risk is still present and could be soccer-specific. Amyotroph Lateral Scler. (2009) 10:205–9. doi: 10.1080/17482960902721634, 19267274

[40] Raymond J Mehta P Larson T Factor-Litvak P Davis B Horton K. History of vigorous leisure-time physical activity and early onset amyotrophic lateral sclerosis (ALS), data from the national ALS registry: 2010-2018. Amyotroph Lateral Scler Frontotemporal Degener. (2021) 22:535–44. doi: 10.1080/21678421.2021.1910308, 33896281 PMC8559906

[41] Weisskopf MG O'Reilly EJ McCullough ML Calle EE Thun MJ Cudkowicz M . Prospective study of military service and mortality from ALS. Neurology. (2005) 64:32–7. doi: 10.1212/01.WNL.0000148649.17706.D9 15642900

[42] Henriques AR Gromicho M Grosskreutz J Kuzma-Kozakiewicz M Petri S Uysal H . Association of the practice of contact sports with the development of amyotrophic lateral sclerosis. Amyotroph Lateral Scler Frontotemporal Degener. (2023) 24:449–56. doi: 10.1080/21678421.2023.2189911, 36992635

[43] Ramamoorthy D Severson K Ghosh S Sachs K, Answer ALS, Glass JD . Identifying patterns in amyotrophic lateral sclerosis progression from sparse longitudinal data. Nat Comput Sci 2022;2:605–616. doi: 10.1038/s43588-022-00299-w 38177466 PMC10766562

[44] Goutman SA Hardiman O Al-Chalabi A Chio A Savelieff MG Kiernan MC . Recent advances in the diagnosis and prognosis of amyotrophic lateral sclerosis. Lancet Neurol. (2022) 21:35334233:480–93. doi: 10.1016/S1474-4422(21)00465-8 PMC9513753 35334233

[45] Wicks P. The ALS ice bucket challenge - can a splash of water reinvigorate a field? Amyotroph Lateral Scler Frontotemporal Degener. (2014) 15:479–80. doi: 10.3109/21678421.2014.984725, 25431828

[46] Hogden A Foley G Henderson RD James N Aoun SM. Amyotrophic lateral sclerosis: improving care with a multidisciplinary approach. J Multidiscip Healthc. (2017) 10:205–15. doi: 10.2147/JMDH.S134992, 28579792 PMC5446964

[47] Raymond J Berry JD Larson T Horton DK Mehta P. Effects of COVID-19 on motor neuron disease mortality in the United States: a population-based cross-sectional study. Amyotroph Lateral Scler Frontotemporal Degener. (2024) 26:149–56. doi: 10.1080/21678421.2024.2401621, 39276073

[48] Imantalab D Sokhal BS Prasanna Kumar Menon S Kalra S Muller S Mallen C. Demographic trends of motor neurone disease-associated mortality from 1999-2020 in the United States. NIHR Open Res. (2025) 4:79. doi: 10.3310/nihropenres.13786.2 41347232 PMC12673253

[49] de Jongh AD van Eijk RPA Peters SM van Es MA Horemans AMC van der Kooi AJ . Incidence, prevalence, and geographical clustering of motor neuron disease in the Netherlands. Neurology. (2021) 96:e1227–36. doi: 10.1212/WNL.0000000000011467, 33472922 PMC8055340

[50] Chio A Magnani C Oddenino E Tolardo G Schiffer D. Accuracy of death certificate diagnosis of amyotrophic lateral sclerosis. J Epidemiol Community Health. (1992) 46:517–8. doi: 10.1136/jech.46.5.517, 1479322 PMC1059643

[51] Stickler DE Royer JA Hardin JW. Accuracy and usefulness of ICD-10 death certificate coding for the identification of patients with ALS: results from the South Carolina ALS surveillance pilot project. Amyotroph Lateral Scler. (2012) 13:69–73. doi: 10.3109/17482968.2011.614253, 21929354

